# Enhanced anaerobic digestion of dairy wastewater in a granular activated carbon amended sequential batch reactor

**DOI:** 10.1111/gcbb.12947

**Published:** 2022-05-02

**Authors:** Mohanakrishnan Logan, Lea Chua Tan, Corine Orline Nzeteu, Piet N. L. Lens

**Affiliations:** ^1^ National University of Ireland, Galway Galway Ireland

**Keywords:** conductive materials, dairy wastewater, granular activated carbon, methane production, sequential batch reactor

## Abstract

This study investigated the potential of granular activated carbon (GAC) supplementation to enhance anaerobic degradation of dairy wastewater. Two sequential batch reactors (SBRs; 0.8 L working volume), one control and another amended with GAC, were operated at 37°C and 1.5–1.6 m/h upflow velocity for a total of 120 days (four cycles of 30 days each). The methane production at the end of each cycle run increased by about 68%, 503%, 110%, and 125% in the GAC‐amended SBR, compared with the Control SBR. Lipid degradation was faster in the presence of GAC. Conversely, the organic compounds, especially lipids, accumulated in the absence of the conductive material. In addition, a reduction in lag phase duration by 46%–100% was observed at all four cycles in the GAC‐amended SBR. The peak methane yield rate was at least 2 folds higher with GAC addition in all cycles. RNA‐based bacterial analysis revealed enrichment of *Synergistes* (0.8% to 29.2%) and *Geobacter* (0.4% to 11.3%) in the GAC‐amended SBR. *Methanolinea* (85.8%) was the dominant archaea in the biofilm grown on GAC, followed by *Methanosaeta* (11.3%), at RNA level. Overall, this study revealed that GAC supplementation in anaerobic digesters treating dairy wastewater can promote stable and efficient methane production, accelerate lipid degradation and might promote the activity of electroactive microorganisms.

**Nomenclature**
AD: Anaerobic digestion
APHA: American Public Health Association
BMP: Biomethane potential
BOD: Biological oxygen demand
DNA: Deoxyribonucleic acid
cDNA: Complementary deoxyribonucleic acid
CM: Conductive material
COD: Chemical oxygen demand
DIET: Direct interspecies electron transfer
EPS: Extracellular polymeric substances
Eq.: Equation
EU: European Union
FEEM: Fluorescence emission excitation matrix
FOG: Fats, oils, and grease
GAC: Granular activated carbon
GHG: Green house gas
HRT: Hydraulic retention time
IC_50_
: Half‐maximal inhibitory concentration
λ: Lag‐phase time
LCFA: Long‐chain fatty acid
*M*_*o*_
: Maximum methane yield
NMDS: Non‐metric multi‐dimensional scaling
OLR: Organic loading rate
*p*‐value: Probability value
*R*_*m*_
: Peak methane production rate
RNA: Ribonucleic acid
SEM: Scanning electron microscopy
SBR: Sequential batch reactor
TEM: Transmission electron microscopy
*T*_*m*_
: Peak time of biomethane production
TOC: Total organic carbon
SMA: Specific methanogenic activity
TS: Total solid
VFA: Volatile fatty acid
VS: Volatile solid

## 1 INTRODUCTION

Ireland has targeted for a 51% reduction in greenhouse gas (GHG) emissions by 2030 against the 2018 levels with a further aim of a net zero GHG emission by 2050 (Climate Action and Low Carbon Development Act, 2021). One of the ways to achieve this is through the recovery of biogas, where this sector alone has the potential to reduce the worldwide GHG emission by 10–13% (Jain, 2019). Biomethane, that is, upgraded biogas, can save up to 202% of GHG emissions, compared with EU fossil fuels (European Commission, 2017). Therefore, biomethane deployment will contribute to future renewable energy production and help in achieving the targets set out for carbon emission reduction (Scarlat et al., 2018). The anaerobic digestion (AD) technology can help to meet the demand for biomethane, concomitantly managing the issue of waste and wastewater treatment. Ireland has a biogas potential of about a million tonne, but less than 2% of this is currently used (SEAI, 2017). Using waste substrates that are readily available in abundance for AD could help to leverage this biogas potential.

An untapped substrate material for AD is dairy wastewater that has high concentrations of fat, oil and grease (FOG) (Salama et al., 2019). The global dairy industry is expected to grow by 35% by the year 2030 (ICFN, 2018) with Ireland's diary industry ranking as the most profitable in Europe in 2020 (Shalloo et al., 2020). In 2019 alone, 19.5 million tons of liquid milk and 6.4 million tons of cheese were produced, and over 46 billion tons of fresh dairy products were consumed in the European Union (EU) (Shahbandeh, 2020). Due to the growth in production and demand of dairy products, waste/wastewater generated from this process is also increasing. For instance, about 1–2 m^3^ of wastewater is generated per m^3^ of manufactured milk (Paulo et al., 2020). While various characteristics of dairy wastewater (high COD and BOD, proteins, lipids and fat content) makes it favourable for AD (Bella & Rao, 2021), it is also difficult to process due to substrate accumulation and mass transfer limitations caused by the intermediate long‐chain fatty acids (LCFAs). This leads to process failure particularly if no proper strategies are implemented during AD operation. Currently, various practices and strategies are used to deal with dairy wastewater loaded with high LCFA, such as using dissolved air flotation for fat removal prior to AD (Logan et al., 2021); low or intermittent organic loading (Ziels et al., 2017); treatment after a start‐up period with step feeding, that is, sequencing continuous feeding and batch feeding (Cavaleiro et al., 2009); pre‐treatments such as microwave (Zielińska et al., 2013), ozone, or ultrasound (Chen et al., 2021); and/or modified reactor configurations (McAteer et al., 2020). However, these methods are associated with either high operational cost or difficulties with deployment in the existing AD systems.

One of the ways to improve the process is by targeting the acidogenic stage of AD, where LCFAs are slowly degraded into shorter chain volatile fatty acids. This can be done through the application of carbon‐based or metal‐based conductive materials (CMs) such as iron nanoparticles, stainless steel or carbon nanotubes, which have shown evidence of accelerated reaction rates and improved hydrolysis‐acidification and methanogenesis (Liu et al., 2021). In addition, CMs can help to overcome the deterioration of AD from acidification or elevated H_2_ partial pressures (Zhao et al., 2017). Besides, syntrophic activity in microbial communities can be increased in CM amended bioreactors (Zhao et al., 2020).

Current research on CM is mainly performed in batch reactors or experiments feeding with model and simple substrates, whereas investigations on long term pilot‐scale reactor operation with real wastewater are still deficient (Wu et al., 2020). Furthermore, very few studies have used granular activated carbon (GAC) as the CM, targeting treatment of complex wastewaters such as lipid rich wastewaters (Dang et al., 2017; Shrestha et al., 2014). Our previous work showed that biomethane potential assays with GAC supplementation improved lipid (oleate) degradation by 50% and decreased the lag phase time by 1000% (Tan et al., 2021). Notably, Ziels et al. (2017) reported higher microbial bioconversion kinetics and functional stability at pulse feeding rather than continuous feeding of lipid‐rich wastewaters. A discontinuous operation of LCFA accumulation during continuous feeding and subsequent batch degradation of the biomass‐associated substrate achieved an efficient methane production rate (Cavaleiro et al., 2009). Consequently, the evaluation of the long‐term process performance and stability of GAC supplementation to bioreactors treating high strength, unprocessed and fat‐rich dairy wastewater is necessary. In addition, the linkage between microbial activity and physiological changes in the presence of CMs has not yet been evaluated. Hence, this study investigated the enhancement of anaerobic degradation of dairy wastewater with GAC supplementation in a sequential batch reactor (SBR). The effect of GAC addition on the microbial community composition was studied as well.

## 2 MATERIALS AND METHODS

### 2.1 Inoculum, substrate and GAC

The GAC (Alfa Aesar™ Carbon, Norit ROW) was purchased from Fisher Scientific (Dublin, Ireland). The GAC was rod shaped with an average diameter and length of 0.8 and 3 mm, respectively. Prior to use, GAC was soaked in demiwater overnight to remove any particulate carbon attached to the GAC and subsequently air‐dried. GAC was provided at 2 g/L as optimized previously (Tan et al., 2021).

Anaerobic granular sludge collected from a full‐scale AD plant (Kilconnell, Galway, Ireland) treating dairy wastewater at ambient temperature (3–19°C) was used as the inoculum. The sludge was crushed to provide better contact between substrate, GAC and microorganisms. A sludge concentration of 5 g VS/L with a total solid content of 62.5 g TS/kg wet sludge and a volatile to total solid percentage of 89% was used. Dairy wastewater was also collected from the same location as the inoculum (Kilconnell, Galway, Ireland). When not in use, inoculum and dairy wastewater were kept at 4°C. Table 1 shows the characteristics of the dairy wastewater fed to the SBR.

**TABLE 1:** Characteristics of the dairy wastewater fed to the sequential batch reactors

Composition	Unit	Amount
Total COD	mg COD/L	5040 (±366)
Soluble COD	mg COD/L	134 (±22)
Carbohydrate^a^	mg COD/L	28 (±2)
Protein^b^	mg COD/L	37 (±4)
Lipids^c^	mg COD/L	4800 (±310)
Ammonium	mg N/L	41 (±10)
pH	—	7.17 (±0)
Conductivity	mS/cm	3.47 (±0)
TS	g/kg	4 (±0)
VS	g/kg	2 (±0)
VS/TS	%	40 (±0)

^a^ 1.07 g COD/g glucose.

^b^ 1.47 g COD/g BSA.

^c^ 1.26 g COD/g light mineral oil [C_16_H_10_N_2_Na_2_O_7_S_2_].

### 2.2 Sequential bed reactor setup and operation

**FIGURE 1:**
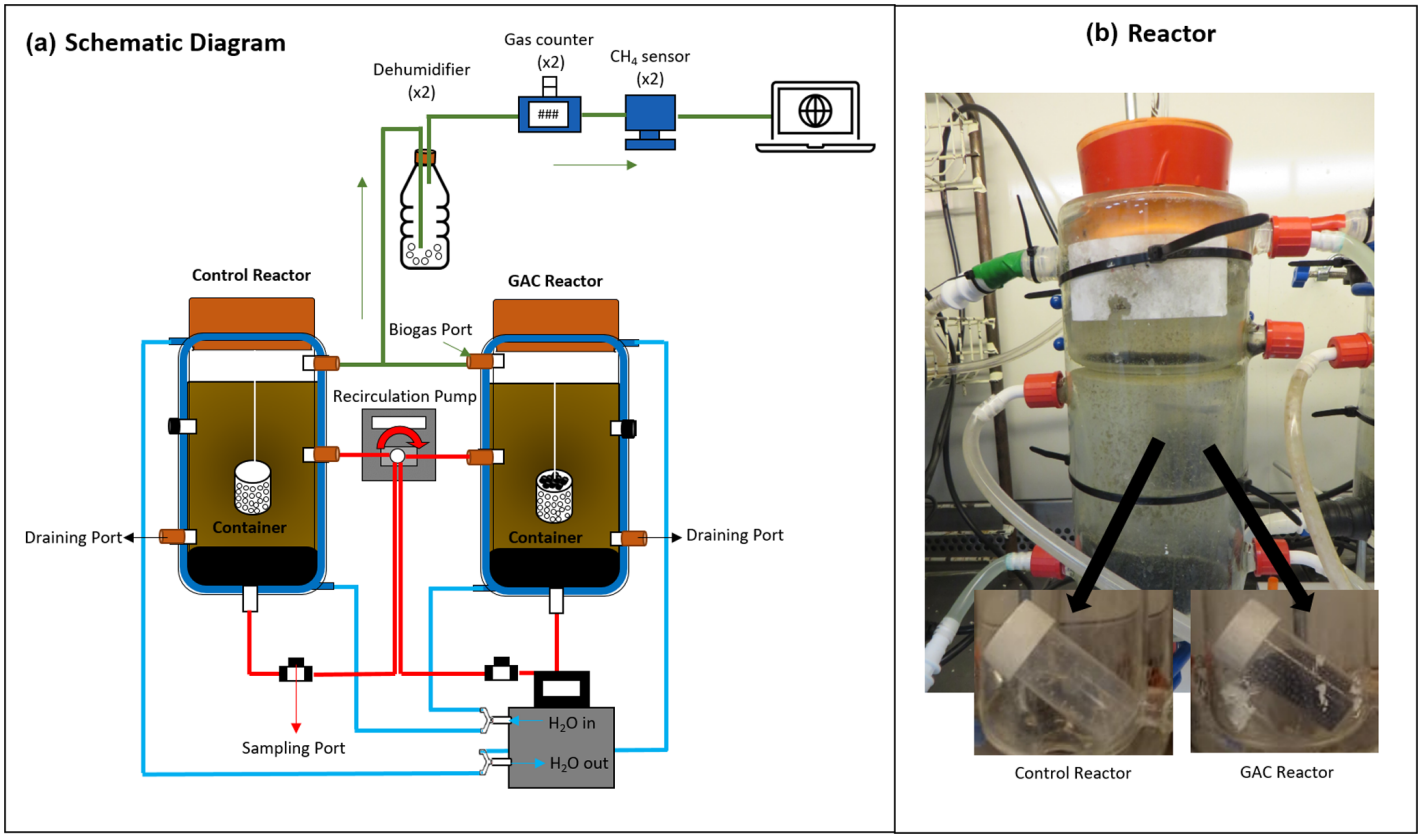
Schematic diagram (a) and image (b) of the UASB reactors (1 L total and 0.8 L working volume) treating dairy wastewater operated in sequential batch cycle mode for a total of 120 days (four cycles of 30 days each) at 37°C and an upflow velocity at 1.5–1.6 m/h. The granular activated carbon (GAC)‐amended reactor was provided with a polypropylene container with 1 mm diameter holes to contain the GAC material (2 g/L). The control reactor was also provided with a similar polypropylene container without GAC

The effect of GAC on the AD reactor treating dairy wastewater was investigated using two 1.0 L double‐jacketed glass upflow anaerobic sludge bed reactors—Control and GAC‐amended SBR—operated in sequential batch mode at a 30‐day operation cycle. Figure 1 shows the schematic diagram of the reactor setup.

The reactors had a total working volume of 0.8 L and an inner diameter of 76.2 mm. The upflow velocity was kept at 1.5–1.6 m/h using a peristaltic pump (Masterflex L/S, Cole‐Parmer, USA). Temperature was maintained at 37°C using a recirculating water bath (Grant Tc120, UK). Biogas lines were connected to the V‐count gas counter and online monitoring CH_4_ sensors (BlueSens, Germany) for measurement of biogas volume and CH_4_ composition, respectively.

A 20 ml polypropylene container was used to contain the GAC material within the GAC‐amended SBR. Holes, approximately 1 mm in diameter, were made on the container using an 18‐gauge needle. This was provided to allow the GAC to have contact with both the wastewater and sludge, while preventing its wash‐out. A similar empty polypropylene container was placed in the Control SBR to ensure that the only difference between the two reactors was the presence of GAC. The container was suspended in the middle of the reactors using a nylon wire attached to the cap and tied to the neopropene rubber stopper used to seal the reactor airtight.

The reactors were operated for a total of 4 cycles, for a duration of 30 days each. Liquid samples were taken from the recirculation line connected to a glass three‐way connector with the sampling port sealed by a septum and aluminum cap. Samples were taken at definite sampling interval points on days 0, 1, 3, 6, 8, 10, 14, 17, 21, 25, and 30 of each cycle run. At the end of the cycle, the recirculation pump was stopped to allow for sludge settling. Approximately 80% of the reactor volume (draining port located near the bottom of the reactor) was drained and new dairy wastewater influent was fed to fill the 0.8 L reactor volume. To ensure anaerobic conditions, a nitrogen gas bag was attached to the headspace line to pump in nitrogen or withdraw headspace gas whenever liquid samples were withdrawn or added. Liquid samples were analyzed for their COD, protein, carbohydrates, NH_4_^+^, VFA, and total lipid concentration. At the end of the operation run (120 days), sludge samples were taken for total solids (TS), volatile solids (VS), extracellular polymeric substances (EPS), and specific methanogenic activity (SMA) analysis.

### 2.3 Analytical method

TS, VS, and total COD were measured according to standard procedures (APHA, 2017). Ammonium and soluble proteins were measured using a Nutrient analyser (Gallery Plus, Thermo Scientific, Waltham, USA). Carbohydrates were measured following the colorimetric protocol described by DuBois et al. (1956) and quantified using a UV‐spectrophotometer (UV‐1900; Shimadzu, Tokyo, Japan).

CH_4_ was converted to its COD equivalent following the ideal gas law and the g COD/g CH_4_ conversion factor is 1.45. Liquid samples for VFA analysis (C_2_ to C_7_) were prepared by adding a known amount and concentration of internal standard (ethyl butyric acid in 10% phosphoric acid) and filtering with a 1740.20 μm filter syringe (Chromafil Xtra Syringe Filters, PET‐20/25). After analysis, VFA concentrations were reported as total VFA COD equivalent. For LCFA analysis (C_10_ to C_18_), samples were prepared by lyophilizing at −56°C and 0.050 mbar for 1 week using a freeze dryer L‐200 basic with Edwards nXDS6iC dry scroll vacuum pump (Buchi, Mason Technology, Dublin, Ireland). After lyophilization, LCFA was extracted and esterified using methanol and hexane following the procedure mentioned by Guihéneuf et al. (2015) with modification. The detailed process for the LCFA extraction is described in Tan et al. (2021).

Total lipid quantification was carried out using Wilks Infracal 2 HATR/ATR‐SP (Hach, Loveland, USA). Prior to total lipid quantification, 4 ml of the liquid sample were taken and lypolized. After lypolization, recovered powder was dissolved in hexane and quantified. The amount of lipids was reported in its COD equivalent following the conversion factor 1.26 g COD/g light mineral oil [C_16_H_10_N_2_Na_2_O_7_S_2_]. Light mineral oil (provided by the manufacturer) was used as the standard for the calibration of a Wilks Infracal 2 HATR/ATR‐SP (Hach, Loveland, USA).

An SMA assay was performed using the sludge sampled from the Control and GAC‐amended SBRs at the end of the fourth cycle (day 120) with acetate (30 mM) as the substrate following the procedure described by Colleran et al. (1992). The total volume of the bottles was 120 ml with a working volume of 20 ml. The anaerobic buffer was prepared with 0.4 mg/L resazurin and 3.05 g/L sodium bicarbonate. The amount of sludge added in each bottle was 2 g VS/L. L‐cysteine hydrochloride (3.2 mM) was used as the reducing agent.

Loosely bound EPS was extracted from a 15 ml sample of the inoculum and both reactor sludges at the end of operation (120 days) through centrifugation at a speed of 10,000 *g* and 4°C for 20 min (Mal et al., 2017). Extracted EPS was quantified for TOC content and normalized at 10 mg/L. Fluorescence emission excitation matrix (FEEM) spectra were recorded on a Shimadzu RF‐6000 (Kyoto, Japan), set to scan samples from 200 to 550 nm (excitation and emission wavelengths) at 6000 nm min^−1^ with an excitation and emission bandwidth of 3.0 nm.

TEM images of the suspensions from both reactors and SEM images of GAC were recorded at the end of 120 days of operation. The protocol followed for TEM and SEM preparation, fixation and imaging were described in detail by, respectively, Florentino et al. (2020) and Tan and Lens (2021).

### 2.4 Microbial community analyses

Approximately 15 ml of sludge samples were taken at the end of each cycle run for microbial community analysis (DNA and rRNA) for further rRNA sequencing for taxonomic characterization of microbial communities. In addition to suspended sludge samples, the biofilm grown in the GAC at the end of the operational run was also harvested and analysed for its microbial community. After sampling, the samples were immediately centrifuged at 8000× *g* for 15 min. The resulting pellet was re‐suspended in a 2 ml RNA tube for 5 h and centrifuged at 10,000 *g* for 10 min. The supernatant was discarded and the pellet was flash‐frozen using liquid nitrogen before storing at −80°C. The pellets were used for RNA and DNA extractions based on the method described by Thorn et al. (2018). RNA purification was carried out using the TURBO DNA‐free™ Kit (Ambion, Dublin, Ireland) in accordance with the manufacturer's instructions. Complementary deoxyribonucleic acid (cDNA) was generated from DNA‐free RNA samples using the superscript reverse transcriptase III kit (Invitrogen, Dublin, Ireland) with random hexamer primers following the manufacturer's instructions.

Both DNA and cDNA were purified using sodium acetate precipitation (Thermo Fisher Scientific, Dublin, Ireland). The purified DNA and cDNA were normalized to a final concentration of 20 ng μl^−1^ and sent to an external laboratory (RTL Genomics, Texas, USA) for *16S rRNA* amplicon sequencing using the MiSeq Illumina platform. In brief, after denoizing and chimera checking, the sequences were clustered into OTUs using the UPARSE algorithm (Edgar, 2013). The centroid sequence from each cluster was then run against either the USEARCH global alignment algorithm or the Ribosomal Database Project (RDP) Classifier against a database of high‐quality sequences derived from the National Center for Biotechnology Information (NCBI) database. The output was then analyzed using the python program that assigns taxonomic information to each sequence. The minimum number of reads was on average of 10,000 reads per sample. The primers used to sequence the V3–V4 region of the bacteria *16S rRNA* genes at both DNA and cDNA level were 357wF (CCTACGGGNGGCWGCAG) and 806R (GGACTACHVGGGTWTCTAAT) (Lemons et al., 2017). Similarly, the V4–V5 region of the archaeal *16S rRNA* genes was sequenced by the universal primary set for archaea: 517F (GCYTAAAGSRNCCGTAGC) and 909R (TTTCAGYCTTGCGRCCGTAC) at both DNA and cDNA level (Florentino et al., 2019). RTL Genomics' data analysis and methodology can be accessed in the following link: http://www.rtlgenomics.com/docs/Data_Analysis_Methodology.pdf. The detailed protocol can also be found in previous literature (Loganathachetti et al., 2016; Pérez‐Rangel et al., 2021; Tianero et al., 2015).

### 2.5 Calculations and statistical analyses

Methane production data were fitted to the modified Gompertz model as described by Logan et al. (2021). Analysis of variance (ANOVA) and the kinetic fitting using the modified Gompertz model were performed using SPSS 16 software. Non‐metric multi‐dimensional scaling (NMDS) analyses applying the Bray–Curtis similarity index was performed by means of the R package “vegan.” The analysis carried out plots the rank order of similarity of DNA‐ and RNA‐based bacterial and archaeal community profiles at the end of different cycles of the Control and GAC‐amended SBRs (Ziganshin et al., 2013). NMDS ordination positions each sample as a function of its distance from all other data points (greater distances represent larger dissimilarities) (Joyce et al., 2018).

## 3 RESULTS

### 3.1 Process performance of granular activated carbon‐amended sequential batch reactor

**FIGURE 2:**
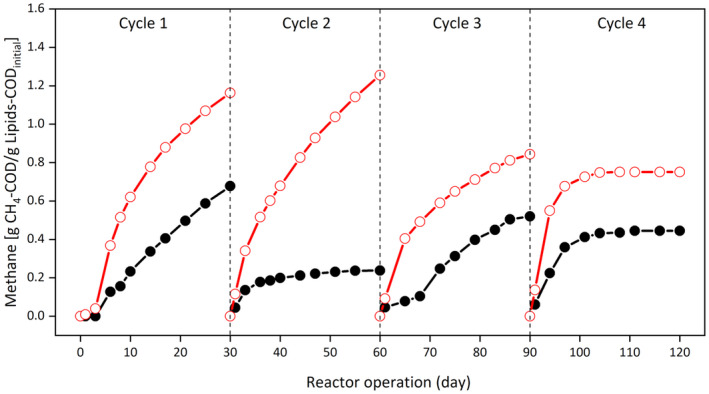
Methane production at all four cycles in the control and granular activated carbon (GAC)‐amended sequential batch reator (SBR). The profile of the control SBR is represented by the black line with solid circle, whereas GAC‐amended SBR is represented by the red line with open circle. Chemical oxygen demand (COD) fraction converted to methane (CH_4_) was measured and converted to its COD equivalent following the ideal gas law (PV = nRT) and the conversion factor 1.45 g COD/g CH_4_

The methane production profile per 30‐day cycle run for both the Control and GAC‐amended SBRs is presented in Figure 2. The methane production at the end of the first 30‐day cycle run was about 68% higher for the GAC‐amended SBR compared with the Control SBR. The highest methane yield of around 1.26 g per g initial COD was realized at the second cycle run in the GAC‐amended SBR, which was about 503% higher than the Control SBR (g COD/g CH_4_ conversion factor = 1.45). The GAC‐amended SBR also showed a 110% and 125% increment in methane yield at the third and fourth 30‐day cycle, compared with the Control. The methane and carbon dioxide concentrations in the biogas ranged between 70% and 80%, and 10% and 20%, respectively, throughout the experimental period.

**FIGURE 3:**
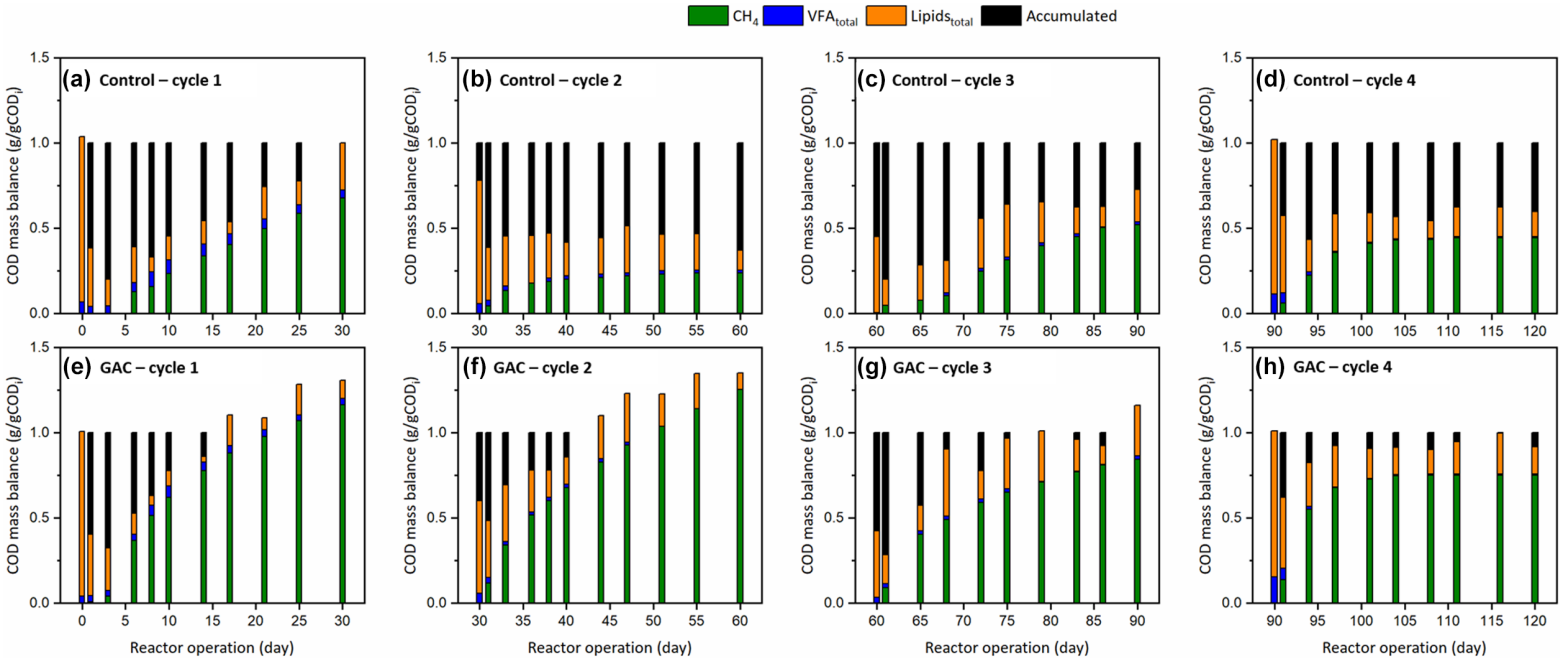
Chemical oxygen demand (COD) mass balance for each 30‐day cycle run for the control (a–d) and granular activated carbon (GAC)‐amended (e–h) reactor. COD fraction converted to methane (CH_4_), total lipids, and total volatile fatty acid (VFA) were measured, quantified and converted to their COD equivalent for each sampling point. COD accumulated was calculated from subtracting all measured values (CH_4_, total lipids, and total VFA) from the initial COD input

The COD mass balance for each 30‐day cycle for both the Control and GAC‐amended SBR are presented in Figure 3. The COD fraction converted to methane was more than 75% at all cycles in the GAC‐amended SBR. The lipid degradation was more profound in the GAC‐amended SBR (Figure 3e–h) than in the Control SBR (Figure 3a–d). More than 50% of the COD, mostly in the form of lipids which might deteriorate the AD performance, accumulated in the Control SBR. Only very low LCFA concentrations could be detected using the analytical methods used (data not shown). Accurate sampling and measurement of the LCFA concentration remains difficult as LCFAs are likely to accumulate in the solid phase, particularly palmitate, which is the limiting step product in LCFA degradation, and is adsorbed onto the biomass matrix (Neves et al., 2009). Samples taken from the effluent thus only represent a fraction of the LCFA concentration. The challenge lies in the extraction of the LCFAs from the solid phase, which is not practical in a semi‐continuous reactor setting.

The pH of the mixed liquor at the start of each cycle was around 7.35 (± 0.15), which decreased to 7.1 (± 0.24) at the end of each cycle for both reactors. The carbohydrate and protein concentrations in the effluent of each cycle were less than 100 mg/L COD in both SBRs, with ammonium reaching a maximum concentration of 120 mg/L N. The TS content of the Control SBR was 15.7 (±1.3) g TS/g wet sludge with 73 (±2)% VS/TS, whereas a reduced TS content of 11.5 (±1.2) g TS/g wet sludge and 74 (±2)% VS/TS was observed in the GAC‐amended SBR at the end of the experimental run.

The kinetic parameters (*R* ^2^ > 0.98) for each cycle of reactor operation estimated by the modified Gompertz model is presented in Table 2. The peak methane yield rate (*R*_max_) was at least two folds higher with GAC augmentation in all the cycles. The highest peak methane yield rate of 0.22 g CH_4_‐COD/g COD‐d was observed at the fourth cycle run in the GAC‐amended SBR. The lag phase duration reduced by 46%–100% in the GAC‐amended SBR, compared with the Control. The sludge taken at the end of the operation of the GAC‐amended SBR had an SMA of 167.9 (± 10.4) ml CH_4_/g VS‐d, which was 36% higher than that of the control SBR.

**TABLE 2:** Specific methanogenic activity (SMA) of sludge taken at the end of the reactor run and calculated kinetic parameters from the modified Gompertz method from each cycle of reactor operation. Kinetic parameters are CH_4_ yield_max_—Maximum methane yield; *λ*—Lag‐phase duration; *R*_max_—Peak methane yield rate and *T*_max_—Peak time of methane yield

		SMA	CH_4_ yield_max_	*λ*	*R*_max_	*T*_max_	*R* ^2^
		Ml CH_4_/gVS‐d	gCH_4_‐COD/gCOD_i_	d	gCH_4_‐COD/gCOD_i_‐d	d	
Control SBR	Cycle 1	123.2 (±6.3)	0.8	3.3	0.03	12.6	0.9891
	Cycle 2		0.2	0.3	0.03	3.0	0.9839
	Cycle 3		0.6	3.0	0.03	9.9	0.9813
	Cycle 4		0.5	0.5	0.05	3.8	0.9824
GAC‐amended SBR	Cycle 1	167.9 (±10.4)	1.1	1.8	0.07	7.6	0.9813
	Cycle 2		1.2	0.0	0.06	7.6	0.9895
	Cycle 3		0.8	0.0	0.06	5.0	0.9841
	Cycle 4		0.8	0.2	0.11	2.7	0.9936

### 3.2 Sludge characterization from control and GAC supplemented SBR

**FIGURE 4:**
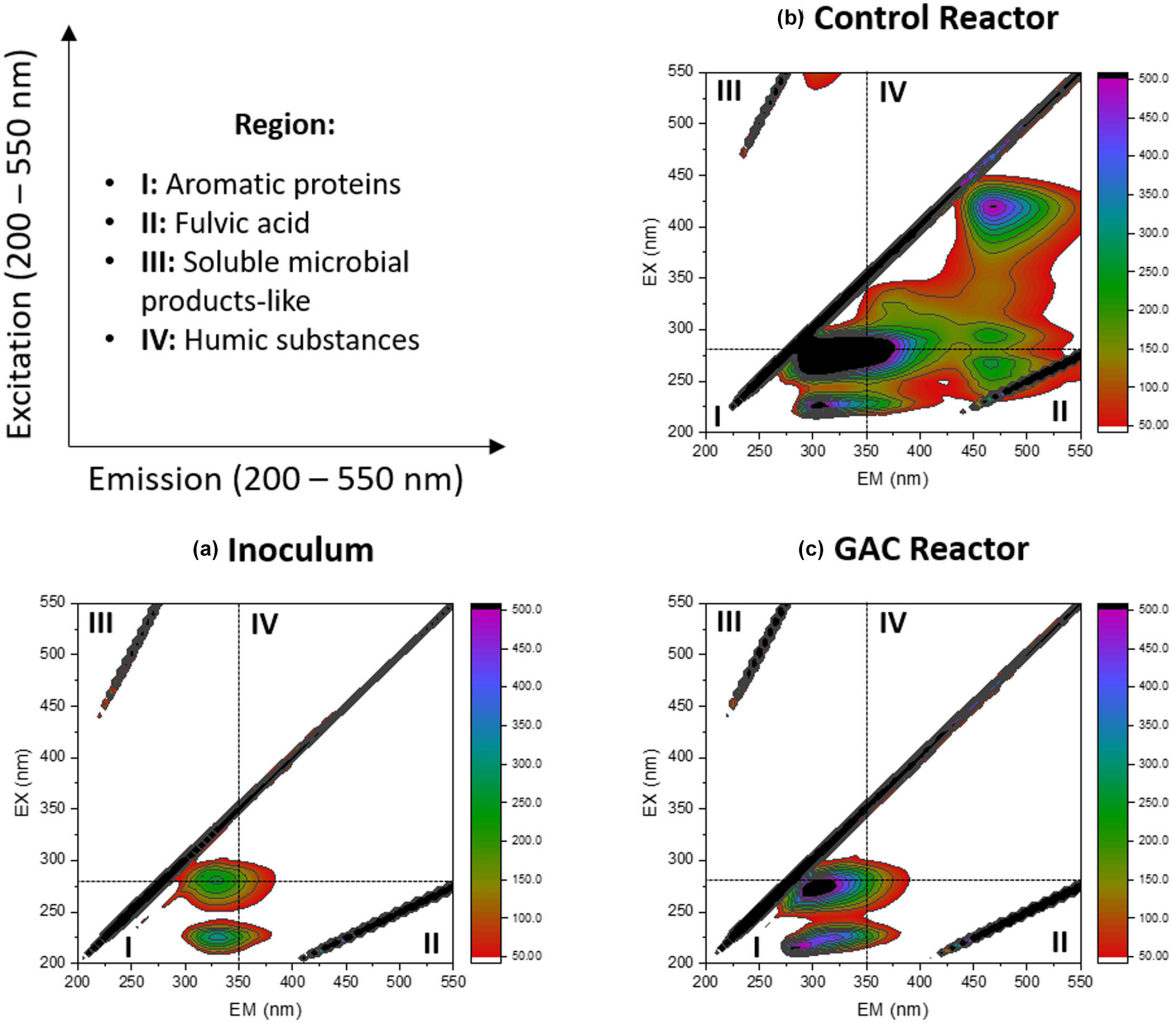
Fluorescence emission excitation matrix (FEEM) characterization analyses of extracellular polymeric substances extracted from the initial inoculum (a), sludge from the control sequential batch rector (SBR) after 120 days or end of Cycle 4 (b) and sludge from the granular activated carbon–amended SBR after 120 days or end of cycle 4 (c). Prior to FEEM analyses, the total organic carbon (TOC) content was normalized to 10 mg/L

FEEM characterization revealed that the EPS extracted from the sludge at the end of the Control SBR operation contained high amounts of TOC that consisted of aromatic proteins, fulvic acid and humic substances with low indication of soluble microbial product‐like compounds (Figure 4b). Opposite to this, the EPS extracted from the sludge of the GAC‐amended SBR showed a low TOC concentration with mainly aromatic proteins present along with a small percentage of fulvic acid and humic substances (Figure 4c).

**FIGURE 5:**
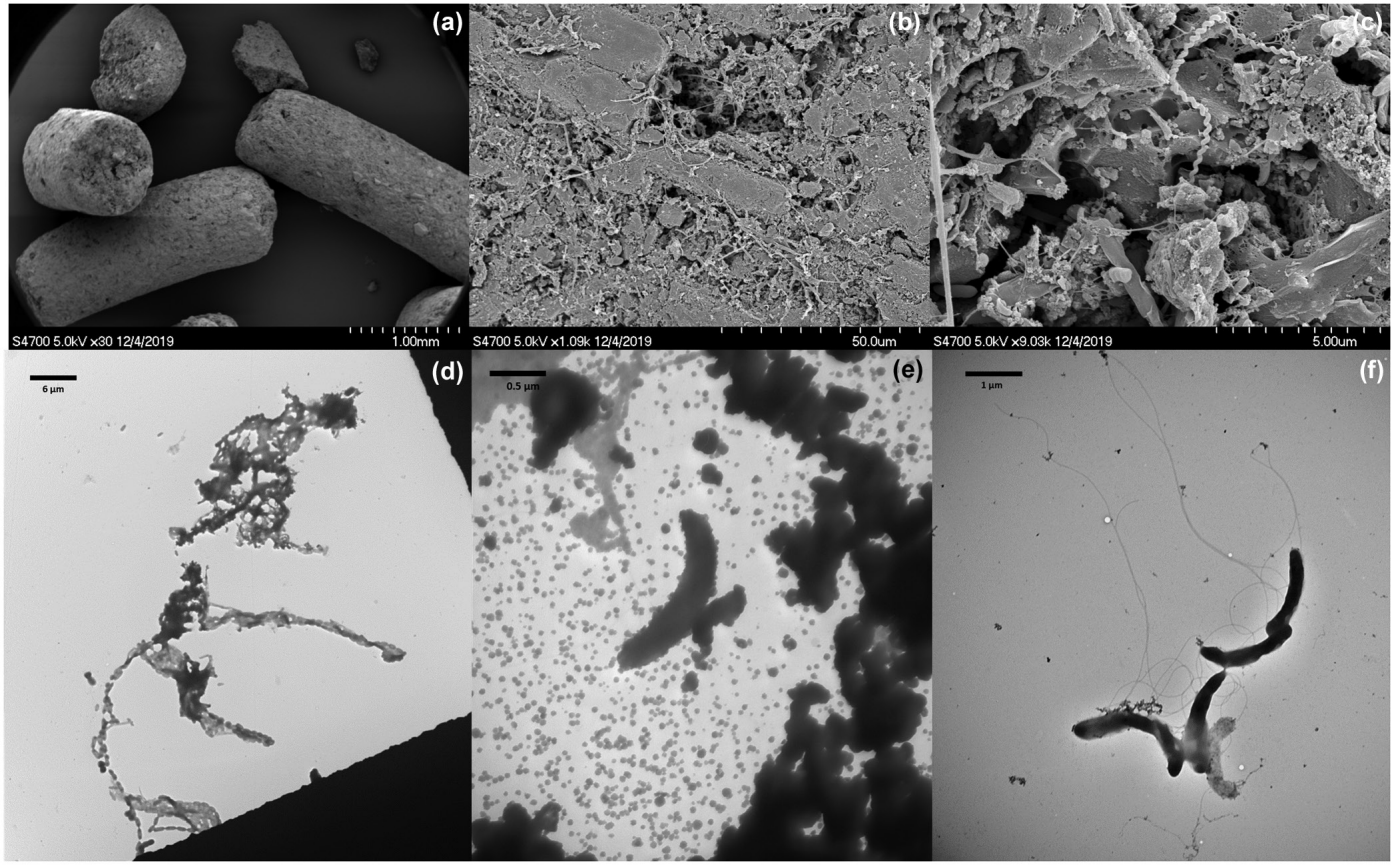
Electron microscopic images. Scanning electron microscopy (SEM) images at different magnification of granular activated carbon (GAC) taken from the GAC‐amended sequential batch reactor (SBR) at the end of 120 days of operation (a–c), as well as transmission electron microscopy (TEM) images of particles present in dairy wastewater (d), suspension of the control SBR mixed liquor (e) and suspension from the GAC‐amended SBR mixed liquor (f). Both suspensions were taken at the end of the reactor run (day 120)

Figure 5 presents the SEM images of GAC taken from the GAC‐amended SBR, along with the TEM images of the suspensions from the Control and GAC‐amended SBR at the end of the reactor operation. SEM images show evidence of biofilm growth on the GAC surface (Figure 5b) with pili‐like structures observed on the attached microorganisms (Figure 5c). TEM images taken from the GAC‐supplemented SBR similarly showed long connecting structures (Figure 5f) between microorganisms that were not evident in the images from the Control SBR (Figure 5e).

### 3.3 Microbial community dynamics

**FIGURE 6:**
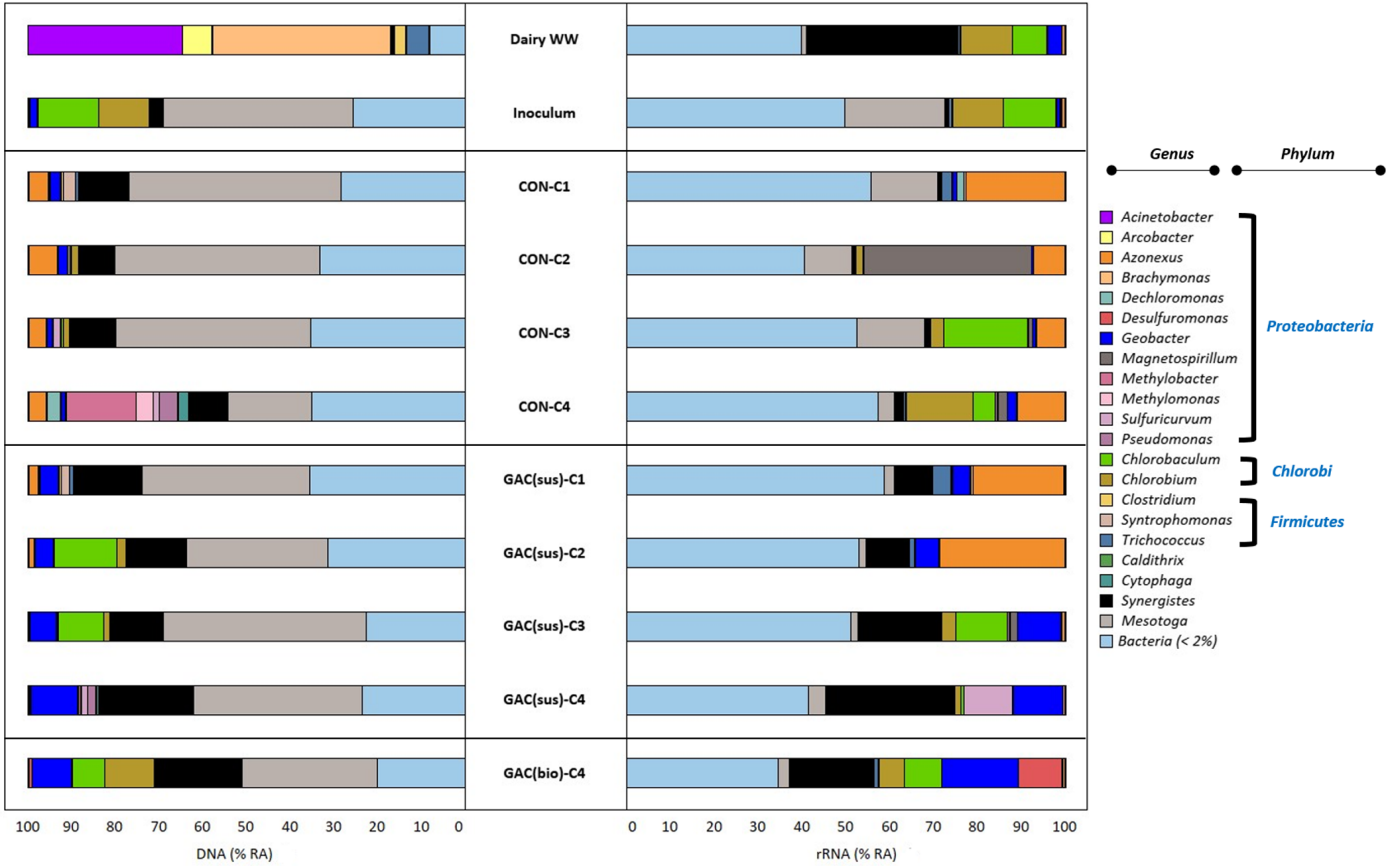
Relative abundance of bacteria at genus level in samples collected from dairy wastewater, inoculum, control and granular activated carbon (GAC)‐amended sequential batch reactor (SBR) (at the end of each cycle run), and biofilm grown in the GAC at the end of the experiment. Genus level with relative abundance lower than 2% were included in unclassified groups. Sample designation labels are as follows: Dairy WW—Dairy wastewater; inoculum—Inoculum used as seed sludge in both SBRs at the start of the operation; CON‐C1, CON‐C2, CON‐C3, CON‐C4 are suspensions from the control SBR after Cycles 1, 2, 3, and 4, respectively; GAC(sus)‐C1, GAC(sus)‐C2, GAC(sus)‐C3, GAC(sus)‐C4 are suspensions from the GAC‐amended SBR after Cycles 1, 2, 3, and 4, respectively; and GAC(bio)‐C4—Biofilm in GAC at the end of the final Cycle 4

**FIGURE 7:**
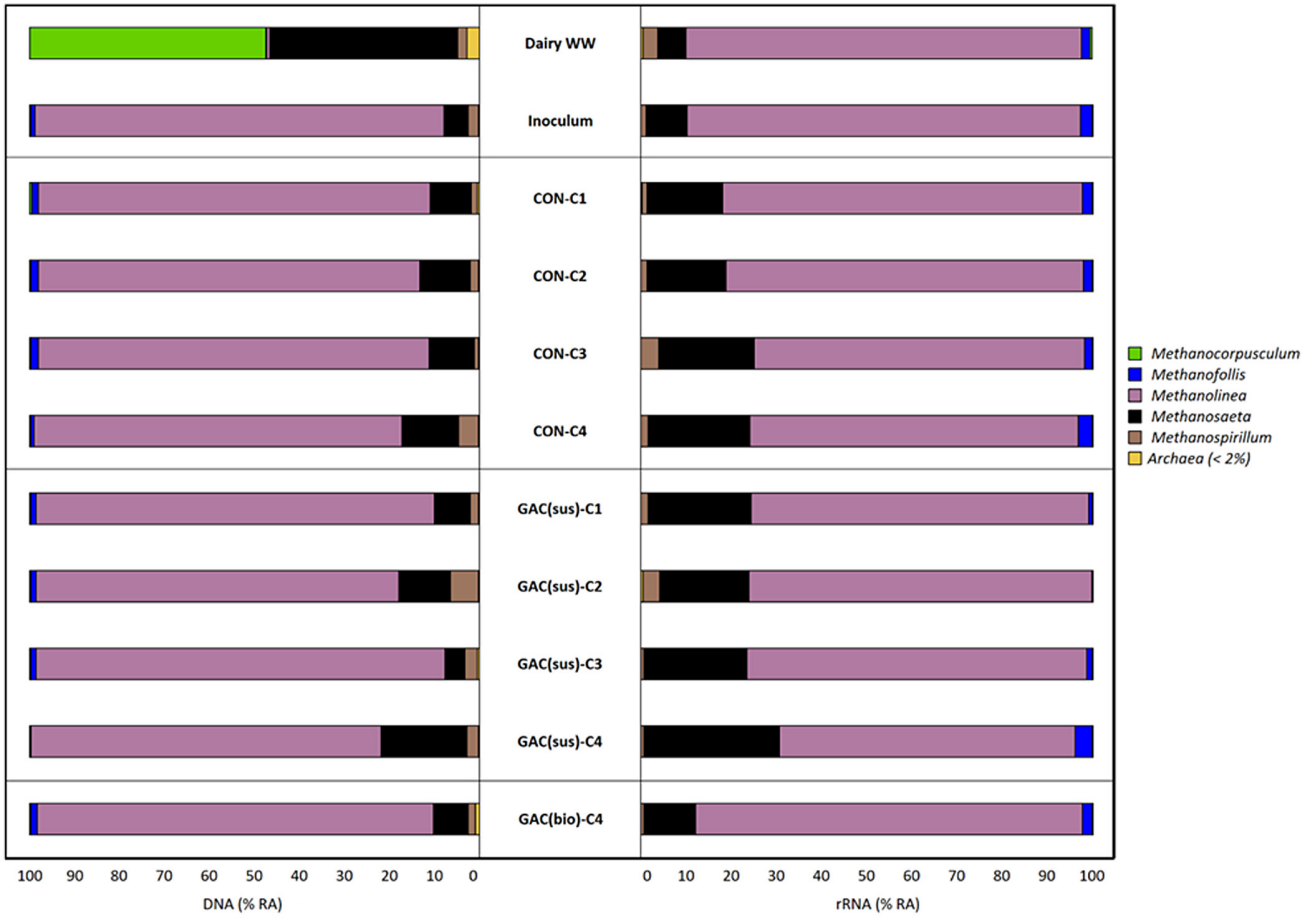
Relative abundance of archaea at genus level in samples collected from dairy wastewater, inoculum, control and granular activated carbon (GAC)‐amended sequential batch reactor (SBR) (at the end of each cycle run), and biofilm grown in the GAC at the end of the operation. Genus level with relative abundance lower than 2% were included in unclassified groups. Description for the sample designations is the same as that of Figure 6

The microbial community structure of sludge growing in the control and the GAC‐amended SBR were investigated by means of *16S rRNA* profiling from DNA and cDNA samples (Figures 6 and 7). The bacterial and archaeal communities present initially in the dairy wastewater and the inoculum that was previously adapted to dairy wastewater are also presented in Figures 6 and 7.

The DNA‐based bacterial communities of the GAC‐biofilm revealed that the main genera were *Mesotoga* (31.1%), *Synergistes* (19.4%), *Chlorobium* (11.1%), *Geobacter* (8.9%), *Chlorobaculum* (7.7%), *Desulfuromonas* (1.0%), and *Trichococcus* (0.5%). *Geobacter* showed a progressively increasing relative abundance growing from 1.1% up to 6.3% at DNA level by the end of the operation. The relative abundance of *Synergistes* also increased from 8.0% to 21.7% at DNA level by the end of the four cycles. Figure 6 shows *Geobacter* and *Synergistes* were enriched in the suspension of the GAC‐amended SBR, relative to the Control SBR, accompanied with a slight enrichment of *Trichococcus*, *Clostridium*, *Deslfuromonas*, and *Caldithrix*.

**FIGURE 8:**
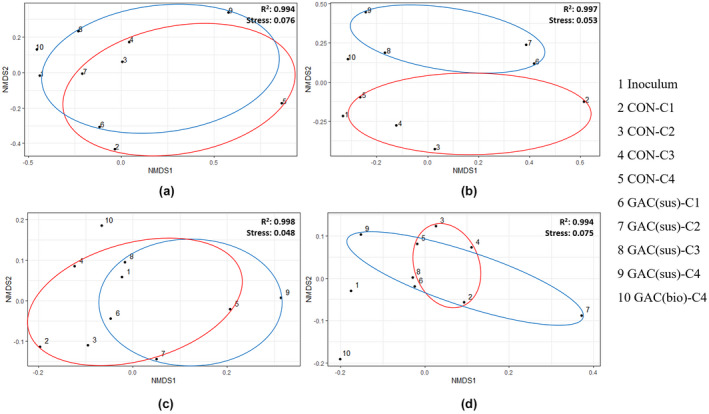
Nonmetric multidimensional scaling (NMDS) ordination with (a) DNA‐based bacterial communities, (b) RNA‐based bacterial communities, (c) DNA‐based archaeal communities and (d) RNA‐based archaeal communities. The Bray–Curtis index was performed to generate NMDS to visualize microbiome similarities. The red and blue cluster denote the control and granular activated carbon (sus) microbiome, respectively. A stress value <0.05 is considered an excellent fit; 0.05–0.1 indicates a good fit; >0.2 indicates a poor fit. Description for the sample designations is the same as that of Figure 6

RNA‐based bacterial analysis revealed similar enrichment of *Synergistes* (0.8% to 29.2%) and *Geobacter* (0.4% to 11.3%) in the GAC‐amended SBR, compared with the Control SBR (Figure 6). A slight increase in *Arcobacter*, *Trichococcus*, *Clostridium*, *Brachymonas*, *Cytophaga*, *Desulfuromonas*, *Sulfuricurvum*, *Acinetobacter*, *Pseudomonas*, *Azonexus*, and *Caldithrix* was observed at cDNA level due to GAC supplementation, relative to the Control. At cDNA level, the biofilm developed on the GAC constituted of *Synergistes* (19%), *Geobacter* (17.5%), *Desulfuromonas* (9.9%), *Chlorobaculum* (8.5%), *Chlorobium* (5.7%), *Mesotoga* (2.7%), and *Azonexus* (0.5%). An NMDS plot stress value below 0.1 indicates that the two‐dimensional representation is ideal for data interpretation (Rees et al., 2004). The NMDS clustering of bacterial communities presence in the Control and GAC‐amended SBR visibly separated at cDNA level (*R* ^2^: 0.997; Stress: 0.053) (Figure 8). In contrast, there was no distinct NMDS clustering at DNA level (*R* ^2^: 0.994; Stress: 0.076).

As depicted in Figure 7, DNA‐based archaeal community analysis shows *Methanolinea* (88.1%), *Methanosaeta* (7.7%), *Methanospirillum* (1.7%), and *Methanofollis* (1.6%) had a remarkable prevalence in the GAC‐biofilm. The relative abundance of *Methanospirillum* increased from 0.9% to 6.3% and *Methanolinea* increased from 86.7% to 91.0% at DNA level in the suspension of the GAC‐amended SBR. The relative abundance of *Methanosaeta* and *Methanospirillum* increased from 16.66% to 22.71% and 1.20% to 1.62% at cDNA level in the GAC‐amended SBR after the first feeding cycle, which was not significantly enriched at the subsequent cycles. *Methanolinea* (85.8%) was the dominant archaea in the biofilm grown on GAC, followed by *Methanosaeta* (11.3%), *Methanofollis* (2.2%), and *Methanospirillum* (0.6%) at cDNA level. The NMDS plot revealed that there was no distinct clustering of the archaeal communities at both DNA (*R* ^2^: 0.998; ctress: 0.048) and RNA (*R* ^2^: 0.994; stress: 0.075) level related to the GAC supplementation. The bacterial and archaeal communities developed in the GAC biofilm were clearly distinct at both DNA and cDNA levels compared with the control‐SBR suspensions (Figure 8a–d).

## 4 DISCUSSION

### 4.1 Improvement of AD of dairy wastewater by GAC supplementation

This study demonstrates GAC supplementation in anaerobic digesters could promote syntrophic metabolism to improve methane recovery from a complex industrial wastewater, that is, dairy wastewater (Figure 2). This study confirms significant improvement in the COD conversion to methane and SMA can be achieved through the addition of GAC during the AD of dairy wastewater (Table 2).

Lipids are rapidly hydrolysed to glycerol and LCFAs, and the LCFAs are subsequently converted to acetate and hydrogen through the β‐oxidation pathway, cleaving 2‐carbon atoms at a time concomitantly with the release of acetyl‐CoA, and finally to methane (Tan & Lens, 2021). Though the LCFA components could not be clearly quantified, it is highly likely that they had either accumulated or were absorbed onto the solid phase, that is, biomass (Neves et al., 2009). These LCFAs might have a detrimental effect by either damaging the cellular membrane of methanogenic bacteria, reducing microbial‐substrate interaction via encapsulation, or inducing flotation via entrapment (Logan et al., 2021). Slow acidification also leads to a prolonged degradation time and extended lag phase duration (Wu et al., 2017). AD of dairy wastewater in the absence of GAC witnessed lipid accumulation in the SBR (Figure 3). However, the supplementation of GAC as CM in AD of dairy wastewater facilitated lipid degradation, with increased lipid degradation and VFA conversion (Figure 3). Therefore, this study shows that GAC amendment could improve both the fermentation and methanogenesis steps.

The lag phase reduction by 100% upon GAC addition (Table 2) indicates that it induced faster methane production from dairy wastewater. Dębowski et al. (2020) evaluated AD of dairy wastewater in a multi‐section horizontal flow reactor equipped with microwave and ultrasonic generators (OLR: 1–4 g COD/L.d; 85% organic removal and 0.23 L/g COD methane yield). This resulted in a decreased productivity at higher OLR. Bella and Rao (2021) showed AD of dairy wastewater can be enhanced by pretreatment or co‐digestion with substrates such as animal manure, agro waste, municipal waste and sewage sludge. A pilot scale magneto‐active hybrid anaerobic biofilm reactor (OLR: 6–8 g COD/L.d; 80% COD removal; 0.26–0.32 L/g COD biogas yield) was demonstrated by Dębowski et al. (2018). Rajesh Banu et al. (2007) evaluated two‐stage hybrid UASB reactors (OLR: 10.7–21.4 g COD/L.d; 98% COD removal; 3.2 m^3^/m^3^ of reactor volume/day biogas yield) for anaerobic treatment of dairy wastewater. Unlike the high cost associated with the aforementioned strategies, faster (reduced lag phase) and improved methane production was relatively easily realized in this study with the addition of GAC. The improvement in the methane yield in the GAC‐amended SBR was more evident in Cycle 1 and Cycle 2, than in Cycle 3 and Cycle 4 (Figure 2). Therefore, it might be possible that the presence of GAC is crucial only during the start‐up period of anaerobic digesters. This could be ascertained through a long term operation of the control and GAC‐amended bioreactors. Notably, other strategies such as step feeding during start‐up could promote sludge acclimation and is conducive to the development of anaerobic microbial communities for efficient LCFA mineralization (Cavaleiro et al., 2009).

Based on the comparison between degradation (Figure 3) and microbial community (Figures 6 and 7) profiles, GAC supplementation could be effective in enriching electroactive microorganisms, overcoming the bottleneck of lipid breakdown. Connection between the enrichment of electroactive species and improved lipid breakdown should be explored further in future works through transcriptomic techniques and analysis.

The inhibition alleviation observed in the GAC amended SBR can be linked to the porosity, conductivity and adsorption properties of GAC. GAC provides a large specific surface area to enhance the growth and proliferation of methanogens (Johnravindar et al., 2020). The electrical conductivity of GAC is reported to be about 3000 S/cm, which is 100% greater than other organic CMs such as biochar (Baek et al., 2018). As such, GAC has a huge potential to serve as an electron conduit, allowing for electron transfer from bacteria to methanogenic archaea. It is noteworthy that high strength wastewaters, such as dairy, improve methanogenesis due to GAC addition, also overcoming the inhibition due to GAC adsorption of low‐strength wastewater (Florentino et al., 2019). Calabrò et al. (2021) also reported that the inhibitory compounds (e.g., LCFAs, VFAs, and alcohols) are adsorbed onto the GAC. The enhanced GAC‐induced methane recovery from dairy wastewater might also be due to improvement in sludge conductivity (Liu et al., 2021) or increased biomass concentration (Guo et al., 2020).

Electroactive bacteria oxidize organics, and then the released electrons are directly transferred through pili to methanogens, which reduce CO_2_ to CH_4_ (Rotaru et al., 2014). The SEM imaging of GAC‐biofilm showed various cell types, suggesting multiple microorganisms are involved in the CM‐induced methanogenesis (Figure 5). The microorganisms with rich pili‐like structures (Figure 5b,c) might facilitate biofilm formation and direct interspecies electron transfer (DIET) (Guo et al., 2020). However, both the quantification and the conductivity of these pili‐like structures needs to be ascertained in future research. Pili deficient strains could not convert ethanol to methane unless in the presence of biochar as CM (Chen et al., 2014), by using 86% of the electrons released from ethanol oxidation for methane production. Further investigations are required to confirm, first, the percentage of the improved DIET process due to GAC supplementation and second, whether the e‐pili concentration increased and assisted in the exogenous electron transfer via GAC acting as the conduit.

The humic substances and redox proteins present in the EPS matrix are widely reported to be electrochemically active and enhance AD (Xiao & Zhao, 2017). FEEM characterization revealed that the EPS composition was not altered in the sludge from the GAC‐amended SBR compared with the Control SBR (Figure 4). Therefore, the improved mechanism with the addition of CM was not via enriched EPS compounds. This contrasts Yan et al. (2018), who found that the presence of CMs greatly enriched certain EPS compounds such as proteins and humic substances that can act as electron shuttles. Notably, higher EPS concentrations in the control sludge without the presence of GAC also suggests the microbes were retained in stress conditions induced from lipid accumulation. Conversely, GAC amendment could have alleviated this stress condition with improved electron transfer and reduced LCFA toxicity, thereby showing less EPS compounds (Figure 4).

### 4.2 Effect of granular activated carbon addition on microbial community composition

Improved lipid degradation to methane is accompanied by the enrichment of fermentative bacteria (such as *Synergistes* and *Geobacter*) and methanogenic archaea (such as *Methanolinea* and *Methanosaeta*) that could possibly establish syntrophic relationships in the presence of GAC (Figures 6 and 7). Kang et al. (2019) also reported that GAC significantly enriched *Geobacter* species. Bioaugmentation of syntrophic microorganisms such as *Geobacter* and *Synergistes* identified in this study could be evaluated in future studies on enhanced AD process performance (Zhang et al., 2018).

Interestingly, the biofilm developed on the GAC had a remarkable presence of electrotrophic methanogenic archaea, especially *Methanolinea* and *Methanosaeta* (Figure 7). Both *Methanolinea and Methanosaeta* are reported as syntrophic partners of *Geobacter* (Jiang et al., 2020; Lee et al., 2016; Mei et al., 2018; Yang et al., 2019). Looking closely at the organisms enriched in the reactor, *Geobacter* and *Methanosaeta* were identified to perform DIET using pili to shuttle electrons (Venkiteshwaran et al., 2016). The cells of these syntrophic partners attach to the carbon‐based materials for interspecies electron exchange since the CMs could save cell energy to produce extracellular electrical connections (Zhao et al., 2015). Pyrosequencing of *16S rRNA* genes from the biomass attached to GAC by Lee et al. (2016) also demonstrated the enrichment of the exoelectrogen *Geobacter* and hydrogenotrophic methanogen *Methanolinea*. *Geobacter* species form electrically conductive aggregates composed of c‐type cytochromes and conductive type IV pili (Aulenta et al., 2020).

Zhang, Zhang, et al. (2020) states that GAC stimulated the e‐pili gene expression in the AD reactor, leading to a higher pili production, thereby increasing the electron transfer efficiency and thus syntrophic methanogenesis. Further investigation is required to show evidence of e‐pili genes in the GAC‐amended SBR through metagenomics and transcriptomic quantification (Holmes et al., 2017). The evaluation of the conductivity of the e‐pili observed due to GAC supplementation by two‐probe or four‐probe conductivity methods is also required (Lovley, 2017).

Nakasaki et al. (2019) stated that *Synergistes* bacteria enhance the lipid degradation through active acetate degradation, pulling the degradation system forward. Similarly, some species in the *Geobacter* genus showed existence of genes encoding a long‐chain fatty acyl‐CoA dehydrogenase, FadE, suggesting the potential for LCFA metabolism (Cavaleiro et al., 2020). In addition to *Geobacter* and *Synergistes*, the presence and activity of LCFAs degrading bacteria such as *Acinetobacter*, *Arcobacter*, *Azonexus*, *Syntrophomonas*, *Pseudomonas*, and *Clostridium* were observed due to the presence of GAC (Baserba et al., 2012; Ning et al., 2018; Westerholm & Schnürer, 2019; Wongfaed et al., 2020; Zhu et al., 2015). Likewise, *Methanospirillum* has been proposed as important hydrogen‐using partner for LCFA‐degrading bacteria, while *Methanosaeta* is reported to be tolerant to LCFAs (Amha et al., 2017; Treu et al., 2016). The microbial community evolution in this study could further explain how GAC supplementation resulted in an effective utilization of the LCFAs, shortening the bottleneck of long lipid degradation lag phase.

Zhang and Lu (2016) reported on conductive ferrosoferric oxide (Fe_3_O_4_) nanoparticles accelerating syntrophic methane production from butyrate oxidation in lake sediments. Guo et al. (2020) found a syntrophic partnership between propionate‐oxidizing bacteria and methanogenic archaea in the presence of CM. Zhang, Guo, et al. (2020) reported that GAC amendment improved conversion of propionate to methane by 100% at a high H_2_ partial pressure (0.17 atm). Zhao et al. (2017) reported that carbon‐based CM enhanced the resistance of semi‐continuous digesters treating butanol to acidic impacts and maintained stable methanogenesis. *Synergistes* bacteria especially use acetate through syntrophic acetate oxidation coupled with hydrogenotrophic methanogens (Ito et al., 2011). This clearly explains the reason for effective VFA conversion in the AD systems amended with CMs (Kumar et al., 2021).

Several studies report that CM supplementation has introduced only small changes in the microbial community composition without changing the dominant microorganisms (Guo et al., 2020; Zhang, Guo, et al., 2020). Van Steendam et al. (2019) asserted that the RNA‐based community analysis is essential to understand the effects of GAC on DIET performing microorganisms. Further, Guo et al. (2020) stated that DNA‐based communities are not significantly different between the reactors with and without GAC supplementation. DNA microbial community analysis in our earlier batch study also revealed similar relative abundance in GAC supplemented and control assays with the dominant bacterial families *Clostridiacea*, *Synergistaceae* and the archaea family *Methanomicrobiaceae* (Tan & Lens, 2021). In this study too, no distinct NMDS clustering was visualized after GAC amendment in bacterial communities at DNA level and archaeal communities at DNA and cDNA levels (Figure 8). In contrast, the NMDS clustering of bacterial communities was dissimilar at cDNA level with and without the presence of GAC. Further studies focused on metatranscriptomic and metaproteomic profiles of DIET‐mediated AD mixed cultures are suggested to further establish if the GAC amendment induces changes in the functional phylogenetic members.

### 4.3 Practical aspects of granular activated carbon amendment for efficient dairy wastewater AD

This study suggests that GAC amendment significantly improved AD of dairy wastewater which could be attributed to several possible reasons as discussed above including the enhancement of DIET process which needs to be confirmed using advanced analytical tools, such as microbial (fluorescence in situ hybridization, meta‐omic methods for detecting genes, transcripts and proteins), electrochemical (cyclic voltammetry measurements) and metabolic (carbon isotope analysis and inhibitor tests) characterization methods (Van Steendam et al., 2019). At present, costly and unsustainable strategies are adopted for anaerobic treatment of dairy wastewaters, including pre‐treatments or operation with low OLRs, to overcome process inhibition from LCFAs accumulation and the resulting mass transfer limitation. Dairy wastewaters are generated in large volumes and due to the presence of lipids, a DAF process is used prior to the AD reactor as a standard operating procedure. This is done to remove lipids and prevent complications during digestion as well as allow high loading rates to process the large volume of wastewater produced. Zhang, Guo, et al. (2020) stated that the organic loading rates of industrial wastewater could be increased by GAC amendment. This study confirms a higher organic loading rate of dairy wastewater to AD reactors can be realized with GAC supplementation. Consequently, this study affirms Lee et al. (2016), who suggest CMs addition is an effective approach to increase methane production rates, ultimately reducing the volume of anaerobic digesters.

CMs amended digesters sustain treatment of complex wastewaters and harsh operational conditions (Mostafa et al., 2020). The potential of GAC to act as an electron conduit could be explored to improve mass and electron transfer between substrate‐microbes and bacteria‐archaea, respectively. Existing AD reactors could easily use CMs such as GAC. However, reactor design and modification is necessary in order to prevent CM washout, along with an optimized recirculation flow for better contact between substrate and CMs. Furthermore, GAC regeneration and recycling are not required since biofilms develop on the GAC surfaces, which can help to bio‐regenerate the GAC (Zhang, Guo, et al., 2020). Therefore, a SBR with a submerged GAC encased porous container, as envisaged in this study (Figure 1), could be adopted. Lü et al. (2019) reported that powdered CMs (biochar) doubled the microbial enrichment and increased the methane production from oil by 30%. Thus, formulation of a modified carrier infused with activated carbon could also be considered for the anaerobic treatment of complex wastewaters.

## 5 CONCLUSION

Traditionally, dairy wastewaters abundant in fats and oils have difficulties in achieving high methane recovery due to accumulation and slow degradation of intermediate LCFAs which results in an extended lag phase duration. This study demonstrates that GAC augmentation in a SBR resulted in substantial improvement in performance, whereby a decrease in lag phase duration (46%–100%) and increase in methane production (68%–503%) were observed compared with the control reactor. Faster reduction and removal of the lipid content were evident both visibly and quantitatively in the GAC supplemented reactor, overcoming LCFA inhibition likely due to improved contact between substrate and microorganism. Further metabolic investigation is required to confirm the mechanistic pathway for the improved performance efficiency of the GAC augmented reactor, in particular confirming the occurrence of direct electron interspecies transfer via electrically conductive pili. Active bacterial genera such as Synergistes and Geobacter, as well as the methanogens Methanolinea and Methanosaeta were observed in the presence of GAC. Overall, GAC supplementation via retrofitting of AD reactors treating dairy wastewater shows a huge potential in improving reactor performance and methane recovery leading to a stable and efficient operation.

## CONFLICT OF INTEREST

The authors declare no conflict of interest.

## Data Availability

The Illumina MiSeq sequencing raw data were submitted to the National Center for Biotechnology Information (NCBI) as a BioProject submission with accession number PRJNA826074. All data that support the findings of this study are openly available in Zenodo at 10.5281/zenodo.6429165.
